# Toward a More Comprehensive View of α-Amylase across Decapods Crustaceans

**DOI:** 10.3390/biology10100947

**Published:** 2021-09-22

**Authors:** Leandro Rodríguez-Viera, Daniel Alpízar-Pedraza, Juan Miguel Mancera, Erick Perera

**Affiliations:** 1 Center for Marine Research, University of Havana, Havana 10300, Cuba; 2 Center for Pharmaceuticals Research and Development (CIDEM), Havana 10600, Cuba; daniel.alpizar@cidem.cu; 3 Department of Biology, Faculty of Marine and Environmental Sciences, Instituto Universitario de Investigación Marina (INMAR), Campus de Excelencia Internacional del Mar (CEIMAR), University of Cadiz, 11519 Puerto Real, Cadiz, Spain; juanmiguel.mancera@uca.es; 4 Andalusian Institute of Marine Sciences (ICMAN), Spanish National Research Council (CSIC), 11519 Puerto Real, Cadiz, Spain

**Keywords:** amylase, carbohydrates, crustaceans, decapods, digestion, feeding habits, lobster

## Abstract

**Simple Summary:**

Decapod crustaceans live in practically all marine, freshwater, and semi-terrestrial habitats on Earth, and exhibit a remarkable variation in their feeding behavior, from filter feeding, grazing, and scavenging to hunting. However, most knowledge about digestive biochemistry in crustaceans has come from studies on a few economically relevant species due to the importance of optimized formulated feeds for aquaculture success. Moreover, most data on α-amylases in decapods derived from studies in herbivore and omnivore species. There are few reviews addressing different aspects of the digestive physiology of decapods, including data on digestive enzymes, but no comprehensive review is available on α-amylases in this group and, in general, information on carnivorous species is often neglected. This review summarizes the information obtained on decapods’ α-amylases and uses recent data from a carnivorous lobster as a connecting thread to compare features of α-amylases from species with different feeding habits, drawing a more comprehensive view of the role of α-amylases across decapods crustaceans.

**Abstract:**

Decapod crustaceans are a very diverse group and have evolved to suit a wide variety of diets. Alpha-amylases enzymes, responsible for starch and glycogen digestion, have been more thoroughly studied in herbivore and omnivore than in carnivorous species. We used information on the α-amylase of a carnivorous lobster as a connecting thread to provide a more comprehensive view of α-amylases across decapods crustaceans. Omnivorous crustaceans such as shrimps, crabs, and crayfish present relatively high amylase activity with respect to carnivorous crustaceans. Yet, contradictory results have been obtained and relatively high activity in some carnivores has been suggested to be a remnant trait from ancestor species. Here, we provided information sustaining that high enzyme sequence and overall architecture conservation do not allow high changes in activity, and that differences among species may be more related to number of genes and isoforms, as well as transcriptional and secretion regulation. However, recent evolutionary analyses revealed that positive selection might have also occurred among distant lineages with feeding habits as a selection force. Some biochemical features of decapod α-amylases can be related with habitat or gut conditions, while less clear patterns are observed for other enzyme properties. Likewise, while molt cycle variations in α-amylase activity are rather similar among species, clear relationships between activity and diet shifts through development cannot be always observed. Regarding the adaptation of α-amylase to diet, juveniles seem to exhibit more flexibility than larvae, and it has been described variation in α-amylase activity or number of isoforms due to the source of carbohydrate and its level in diets, especially in omnivore species. In the carnivorous lobster, however, no influence of the type of carbohydrate could be observed. Moreover, lobsters were not able to fine-regulate α-amylase gene expression in spite of large changes in carbohydrate content of diet, while retaining some capacity to adapt α-amylase activity to very low carbohydrate content in the diets. In this review, we raised arguments for the need of more studies on the α-amylases of less studied decapods groups, including carnivorous species which rely more on dietary protein and lipids, to broaden our view of α-amylase in decapods crustaceans.

## 1. Introduction

Decapod crustaceans diverged in the Late Ordovician and most lineages diverged in the Triassic–Jurassic [1]. Since then, this group of animals has experienced a great diversification, and today over 15,000 living species populate marine, freshwater, and semi-terrestrial environments [2]. This ecological success relies, to a great extent, in the capacity of the different groups to adapt to a broad variety of diets. Indeed, decapods exhibit a wide variation in feeding habits, which includes herbivores, carnivores, scavengers, deposit feeders, filter feeders, and opportunistic omnivores [3,4]. In addition, their wide geographic distribution implies that digestion of such a variety of foods occurs over an extensive range of environmental conditions (e.g., temperature, salinity, etc.). After ingestion, digestive enzymes are responsible for the hydrolysis of complex dietary components into assimilable nutrients and accordingly, digestive enzymes harbored by decapods have been studied, although less deeply than in other arthropods such as insects [5].

The digestive enzymes of omnivore crabs [6,7,8,9,10,11] and shrimps [12,13] have been studied from an evolutionary perspective because differences between plants and animals force trade-offs in the traits required to use these feeds simultaneously [14]. Likewise, digestive enzymes adaptation to a vegetarian diet has been studied in different species [15] as at least 31 lineages of marine, freshwater, and terrestrial crustaceans have independently overcome the challenge of consuming plant material [16]. In the case of penaeid shrimps, digestive enzymes studies, and the direct relationship between digestive enzymes and feed utilization, have also been speeded up due to their economic importance in aquaculture worldwide [17]. Conversely, carnivorous species have been historically less studied. However, information has been produced during the last decade on the digestive biochemistry of a carnivorous spiny lobster [18,19,20,21,22,23,24,25], shedding light on aspects such as isoenzyme richness, molecular, and biochemical differences among isoforms, molecular evolution, and regulatory mechanisms.

Crustaceans with particular feeding habits exhibit distinctive digestive enzymes, such as cellulase and hemicellulase in those that feed on leaves [3,26,27,28], or laminarinase in those consuming brown and green phytoplankton and algae [26]. However, all species share main digestive enzymes such as proteases (trypsin, chymotrypsin, etc.), lipases, and α-amylases (α-1,4-alpha-D-glucan glucanohydrolase, EC 3.2.1.1; henceforth named α-amylases). Protein and lipids are well known to be key nutrients for crustaceans metabolism [29,30] while the role of dietary carbohydrates is not that clear and rather variable among species. Even when dietary carbohydrate cannot be efficiently used by aquatic animals [31], carbohydrates are essential and thus included in artificial feeds at 20% to 30% [32,33], although higher carbohydrate intake can lead to slow growth, low immunity, and high mortality rates [31,32]. Among carbohydrases, α-amylase is responsible for the hydrolysis of starch and glycogen, but remained poorly studied in carnivorous decapods until recently [18,19,23,24,25,34]. The new information provided by these recent studies now allows drawing a more comprehensive view of the role of α-amylases across decapods crustaceans.

In this review, we used the information obtained on the α-amylase of a carnivorous lobster, the spiny lobster *Panulirus argus*, during the last decade as a connecting thread to compare features of α-amylases from crustacean decapods with different feeding habits. Although spiny lobsters have sometimes been referred to as omnivores because of the presence of algae or seaweed material in their guts, they are more often considered top predators of benthic communities, and are thus refereed in this review as carnivores. Studies in other species often allowed us to confirm already known trends or provide new insights on poorly understood features of decapods’ α-amylases, while regarding other issues, information is fragmentary and only allowed us to suggest areas where more studies are required for a better understanding of α-amylases in this varied group of animals of economic, ecological, and evolutionary relevance. There are few reviews that address aspects of digestive physiology in decapod crustaceans (e.g., synthesis of digestive enzymes, food processing, nutrient absorption, and metabolism) [11,31,35], but an encompassed analysis of amylases in this group is still incomplete as features of the enzyme from carnivorous species have been somewhat neglected.

## 2. General Features and Activity

Alpha-amylases tertiary structure comprises three distinct domains. The catalytic domain-A ((β/α)8- or TIM-barrel) is the most conserved domain in the α-amylase family, and consists of an amino terminal (β/α) 8- barrel structure [36,37]. In the center of this domain, three residues (Asp, Glu, Asp) form the catalytic site as determined by X-ray crystallography [38] and site directed mutagenesis [39]. B-domain protrudes out of the barrel as a longer loop between the strand β3 and helix α3 and succeeded at the C-terminal end by domain C, adopting an antiparallel β-sandwich fold [40]. The domain C, domain with the lowest degree of conserved sequence, folds into antiparallel β-barrel and forms the C-terminal part of α-amylases [40,41].

Alpha-amylases are calcium metallo-enzymes that act at random locations along the starch chain leading to the hydrolysis of α-1,4 glycoside bonds, and releasing reducing groups in the α-configuration [41,42]. In particular, it produces maltotriose and maltose from amylose, or maltose and glucose, and limits dextrin from amylopectin [42]. The hydrolysis is limited by branches with α1-6 bonds in amylopectin [43]. The rate of hydrolysis depends on the catalytic properties of the enzyme but it is strongly determined by the vegetal origin of the starch [41]. A wide variety of methods have been used for measuring α-amylase activity as recently reviewed. Among them, large differences occur in type and concentration of substrate, hydrolysis products measured, reaction pH and temperature, incubation time, and definition of α-amylase units, though the most common feature is the use of starch as the substrate [44].

## 3. Molecular Features

### 3.1. Gene and Transcript Features

The presence of several α-amylase gene copies may be advantageous for more enzyme production, for fine developmental and tissue-specific expression, for broadening pH and substrate range, or for overcoming the natural defenses of plants if they are included in diet [5]. Molecular information on the α-amylase gene in the crustacean decapod is restricted to few species. In the omnivorous shrimp *Litopenaeus vannamei*, three α-amylase genes have been characterized, with nine introns located at the same positions but presenting no similarity among genes [45]. However, an RNA-seq study found 16 unigenes for α-amylase in this species [46]. Within the Panama natural population, 35 different alleles occur at this locus [45]. In the shrimp *Palaemonetes varians,* population studies found four co-dominant alleles, while some populations only exhibit two of them [47]. In contrast, a single and intron-less gene occurs in the carnivorous lobster *P. argus* [24]. The number of α-amylase genes is also variable in non-decapod crustaceans. For instance, six copies of the α-amylase gene occur in the detritivore isopod *Asellus aquaticus* [48], which eats on leaf material in freshwater environments [49], while two copies occur in another detritivore isopod, *Sphaeroma serratum* [48], which fed on detritus from marine algae or terrestrial plants [49], although its fatty acid signature suggested that animal material is also included in its natural diet. In other arthropods this issue has been studied more thoroughly. In insects, the copy number varies from only 1 (e.g, in honeybees) to more than 12 (in some mosquitoes) [5]. Among them, α-amylase genes have been more thoroughly studied in *Drosophila*, and the number of gene copies within this single genera varies from 1 to 6 [48].

The lobster (*P. argus*) gene encodes a single transcript (PaAmy, GenBank accession no. LK937698) of 1830 bp, with a short 5′ untranslated region of 23 bp, a long 3’ untranslated region of 268 bp, and a 1539 bp ORF. Before the poly A tail, two sites of alternative polyadenylation were found at 108 bp and 139 bp downstream the stop codon. The lobster transcript exhibited high identity with α-amylases cDNAs from other decapods such as *L. vannamei* (79%) and *Penaeus japonicus* (78%) α-amylase, but also high (>60%) with α-amylases from phylogenetically distant groups such as humans (Table 1).

**Figure 1:**
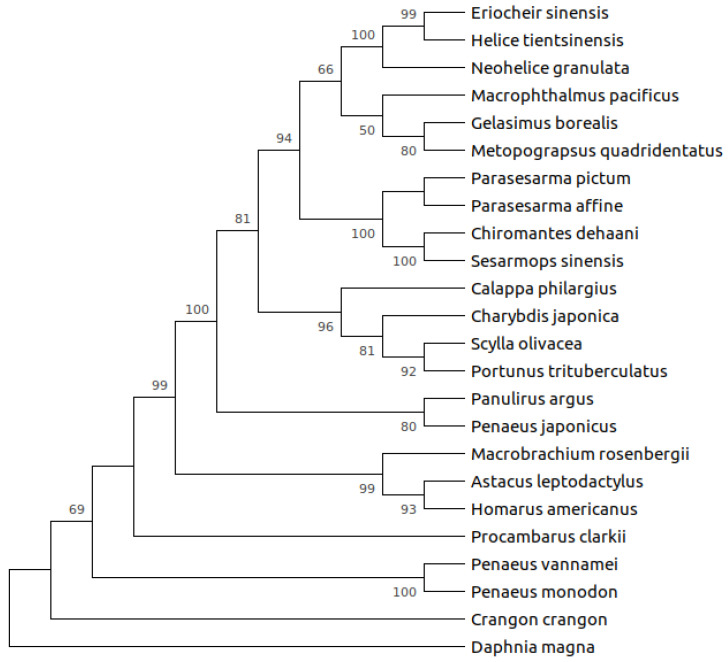
Alpha-amylase diversification is not well understood in decapod crustaceans. Neighbor Joining tree showing phylogenetic relationships among α-amylases from decapod crustaceans. Sequences were aligned using the MUSCLE algorithm. The best-fit model of evolution (TN93 + G + I, gamma shape parameter = 1.01) was selected and the tree was constructed with MEGAX. Topology robustness was tested with 1000 bootstrap replicates. Only bootstrap values higher than 50% are shown as NJ.

There is not a clear and complete picture of α-amylase evolution within decapods crustaceans. A previous phylogenetic analysis including α-amylases from shrimps and lobsters, and those of insects, fishes, amphibians, birds, and mammals, retrieved the expected topology resembling phylogenetic relationships among groups [24]. Within the well-supported Arthropoda clade, crustacean’s α-amylases appeared as a monophyletic group [24]. However, more α-amylase sequences are now available (Table 1), and this allows having a wider view on their sequence evolution, although there are more sequences for crabs than for other groups. Evolutionary analyses of crab’s α-amylases found evidence of positive selection in the enzyme of herbivore crabs, whereas not in omnivore or carnivore species [11]. Nevertheless, a wider analysis, including α-amylases from major groups of decapods crustaceans revealed that while most crab α-amylases appear as a monophyletic group which further diversify, α-amylases from phylogenetically distant groups such as shrimps and lobsters clustered together according to their feeding habits (i.e., carnivores or omnivores) (Figure 1), suggesting that convergent evolution might have occurred among distant lineages with feeding habits as a selection force. Indeed, ongoing analyses at our laboratory revealed that positive selection also occurred at common sites in omnivore species from distant groups such as shrimps and crayfishes. 

### 3.2. Protein Features

**Figure 2:**
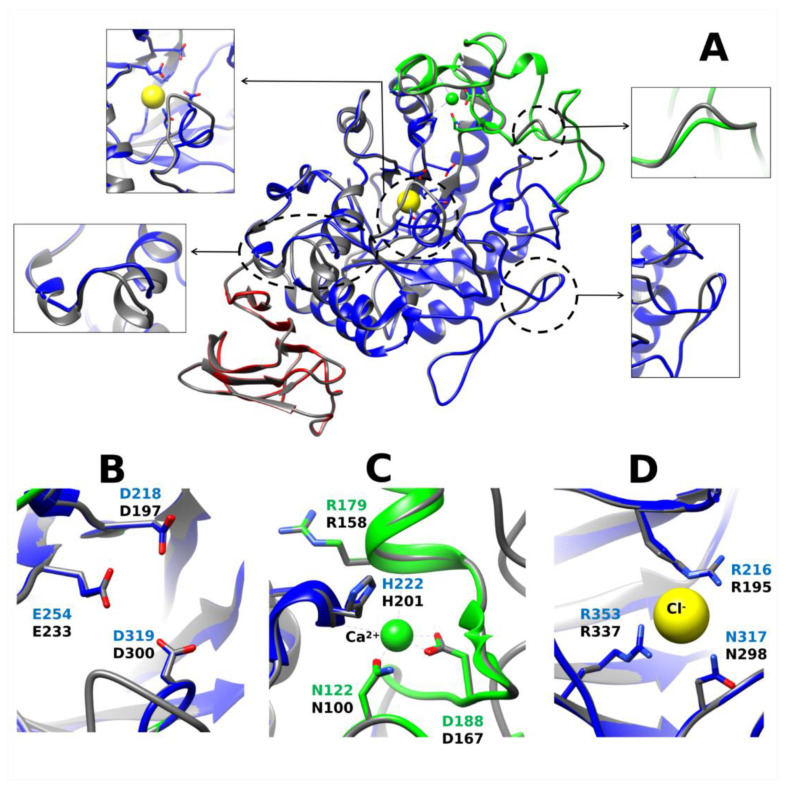
Superimposed structures of *Panulirus argus* α-amylase (PMDB: PM0079556) and human pancreatic α-amylase (gray) (PDB: 1B2Y) (**A**), showing conserved overall architecture. Most notable differences showed in inserts. Three-dimensional structure of the lobster enzyme was predicted by homology modeling [24]. Individual domains and key structural and functional residues are represented in the model. Domain A (the catalytic domain) is shown in blue, domain B in green, and domain C in red. Conformation of residues of the catalytic triad (**B**), and the calcium (**C**) and chloride (**D**) binding sites are predicted to be highly conserved between the lobster and the human α-amylase, with nearly identical geometry. Site numbers start at the first residue of the lobster enzyme including a 21 residues signal peptide not included in the model. Figures were drawn using UCSF Chimera v1.14 (http://www.cgl.ucsf.edu/chimera/), accessed on 10 May 2021.

Alpha-amylase enzymes in decapod crustaceans have estimated molecular weights between 26 and 75 kDa (Table 2). Estimates differ depending on whether they come from electrophoresis mobility or from cDNA sequences. For example, the molecular weight for the lobster *P. argus* α-amylase was estimated to be around 44–47 kDa [18] by electrophoresis whereas 55.5 kDa from its transcript sequence [24]. Few protein sequences for α-amylase of decapod crustaceans are available (Table 3). The lobster transcript encodes a protein with 513 amino acids, including a highly hydrophobic signal peptide of 21 amino acids, a potential cleavage site for the signal peptide between Ala21 and Gln22, and predicted molecular mass and isoelectric point for the mature enzyme of 55.5 kDa and 4.93, respectively. The comparison of amino acid sequence of lobster enzyme and other α-amylases showed a high similarity in conserved regions I to VI, but region VII was not identified. The region VII is known to be less conserved among the family [50]. A model for this α-amylase was developed and deposited at the Protein Model Data Base (http://bioinformatics.cineca.it/PMDB/main.php), accessed on 11 June 2021 under PMDB id: PM0079556. The enzyme has the typical 3D structure of α-amylase enzymes. It is formed by three domains A, B, C. Domain A is a (β/α)8-barrel, B is a loop between the β3 strand and α3 helix of A, and C is the C-terminal extension. PaAmy has the active site cleft between domains A and B, with a triad of catalytic residues (Asp218, Glu255 and Asp319). It contains a calcium-binding site (Asn122, Arg179, Asp194, and His222), a chloride-binding site (Arg216, Asn317, and Arg353), and several cysteines residues (Figure 2A). Ten cysteines residues were observed in the lobster α-amylase, as occur in α-amylases from other arthropods [12,51]. Eight of these cysteines are also conserved in vertebrate α-amylases [52]. The additional two residues in crustaceans and other invertebrates enable a fifth disulfide bridge, and may be related with differences in activity during temperature adaptation [12]. In general, overall architecture of the α-amylase is highly conserved, even when compared with the human enzyme (Figure 2A), although some differences occur in superficial loops which effects on enzyme function are unknown. These effects, if any, may be related with extended interactions with large substrates. Given that these regions are subjected to less evolutionary constrains, their analysis in carnivore, omnivore, and herbivore species may shed light on their evolution across decapod crustaceans, but this examination have been not yet produced. Notably, the geometry of key residues for α-amylase function such as the catalytic triad, and the binding sites for calcium and chloride are highly similar in the lobster and the human enzyme (Figure 2B–D).

## 4. Biochemical Features

Different decapods crustacean α-amylases have been studied with respect to some of their biochemical features. Some groups such as shrimps and crabs, due to their commercial or biological interest, have traditionally been more studied. However, available information is sparse and not homogeneously reported, especially regarding catalytic properties (Table 2). In this section we will focus on different aspects related to biochemical features of α-amylase activity in decapods crustaceans.

### 4.1. Sodium

The α-amylase activity in the marine crab *Maguimithrax spinosissimus* is poorly affected by NaCl [73], although it has been reported that NaCl influences the α-amylase activity in marine shrimps [60], crabs [10], and lobsters [24,55]. For instance, in estuarine amphipod *Gammarus palustris*, activation occurred at low chloride concentrations, achieving 90% of the maximum activity at 8 mM NaCl, but no inhibition occurred at higher concentrations [79]. In the estuarine shrimp *Farfantepenaeus californiensis*, α-amylase activity is highest at a low salt concentration (i.e., 0.01 M NaCl), and it is also poorly affected by high salt concentration, retaining 50% of its activity at 3 M NaCl [60]. Likewise, while α-amylase activity in the euryhaline burrowing crab *Neohelice granulata* is maximal in the wide range of 0.5–1.5 M, it is maintained at high NaCl concentrations (up to 4 M), retaining 30% of initial activity [10]. On the other hand, while in larvae of the marine lobster *H. americanus* α-amylase activity does not significantly vary over the range 0.05–0.2 M NaCl [103], in adult lobsters, activation of the enzyme is highest at 0.1 M NaCl [55], and in the marine spiny lobster *P. argus* at 0.3 M NaCl [24]. In summary, differences occur among the crustacean α-amylases in their response to salt concentration, probably reflecting habitat features and/or evolutionary relationships.

### 4.2. Calcium

At least one calcium binding site occurs in α-amylases [104,105]. Studies in the crabs *Carcinus maenas* [106], *N. granulata* [10], and *M. spinosissimus* [73]; in the spiny lobster *P. argus* [24]; in the crayfish *Cherax quadricarinatus* [70]; in three species of penaeid shrimps [13]; and in other invertebrates [82,86,89] have showed enhancements in α-amylase activity when CaCl_2_ concentration increases up to a maximum and then decreases. However, exceptions occurred, as in the lobster *Homarus americanus*, where no effect of calcium was reported [55], while in the crab *Portunus segnis*, only minor effect of calcium was reported [76]. Calcium binding sites are important structural features of amylase enzymes, and it is well known that this ion is important for the activity and stability of α-amylases. However, the stability effects demonstrated by calcium in decapod amylases are not well understood, and this issue has never been evaluated in many species (Table 2).

### 4.3. pH

Spiny lobster α-amylase activity showed an optimal pH of 4–5 [18] in correspondence to the acidic pH typical of the gastric juice of lobsters [18,34]. Similar features have been reported in the homarid lobsters *H. americanus* (pH 5.2) [55] and the crayfish *C. quadricarinatus* (pH 6.0) [61]. In the spiny lobster, this enzymatic activity is strongly affected at alkaline pH values [18]. Crustacean α-amylases are known to be divided into two groups, one with optimal pH below 6.3 including isopods, amphipods, and Astacura, and other groups with higher pH optimum comprising shrimps and brachyurans [60,74,76,78]. A variety of optimal pH values for α-amylase also occur in insects, another very diverse group of invertebrates. In this way, coleopteran α-amylases have acidic optimum activity and dipteran α-amylases have neutral preference, whereas lepidopteran ones have clear alkaline preference [5]. The role of variation in digesta pH in regulating carbohydrate digestion by α-amylase is not fully understood in crustaceans, and this is a critical point to understand changes in biochemical features reported in α-amylase activity related to pH.

### 4.4. Temperature

In general, the thermal stability of α-amylases is relatively low above 30–37 °C in shrimps [13] and also in some non-decapod species [81]. Although the α-amylase of the tropical king crab *M. spinosissimus* was stable at a high temperature (>50 °C) [73], α-amylase activity from the tropical lobster *P. argus* is compromised above 30 °C [24], as in other crustaceans. Yet, in the crab *P. segnis*, which is tolerant of a wide range of temperatures from 13 °C to 30 °C, the enzyme is also highly stable at 50 °C [76]. Less variation has been observed in optimal temperature, i.e., lobster *P. argus*, 50 °C [24]; shrimp *F. californiensis,* 30–40 °C [60]; and different species of crab, e.g., *Scylla serrata*, 50 °C [74], *N. granulate,* 30–40 °C [10]; and *P. segnis* 50 °C [76]. In summary, the relationship between stability and habitat temperature is not clear, probably because this feature mostly depends on the conserved architecture of the enzyme among crustaceans.

### 4.5. Catalytic Activity

Using CNP-G3 as the substrate, we determined that the lobster *P. argus* α-amylase has *Km* (0.36 mM), which is lower than *Km* of the pancreatic and salivary human α-amylases (1.15 mM) [100]. The *Vmax* of the lobster enzymes is 0.56 ± 0.024 mM mL^−1^ min^−1^, with *Kcat* of 28.42 ± 1.203 s^−1^. This indicates that the lobster enzyme saturates at low substrate concentrations and may be an adaptation of this carnivorous species to low carbohydrate loads after feeding [24]. However, direct comparison on the catalytic properties of different crustacean α-amylases is hampered by the few studies available and the different substrates/methods employed. More often the substrate used is starch, which resembles carbohydrates in formulated feeds and in the natural diet of some herbivore/ omnivore crustaceans, while few studies used glycogen, more present in the natural diet of carnivore crustaceans. One study in the crab *N. granulata* reported lower *Km* for starch than for glycogen (1.24 mg mL^–1^ for starch and 16.19 mg mL^–1^ for glycogen) [10]. However, even using the same substrate results are difficult to compare such as *Km* obtained for α-amylases of *C. maenas* (0.22% starch) [78] and *Garnmarus palustris* (0.04% starch) [79]. The source of starch (e.g., potato, wheat, maize) is also a source of variation. So, in the crab *P. segnis*, *Km* for α-amylase was reported to be 7.5 mg mL^−^^1^ for potato starch [76] but the catalytic activity was lower toward other starches.

With the few studies available and the disparity in methodologies employed for the kinetic determination of α-amylases in decapods, results do not allow the drawing of clear relationships between catalytic activities and other characteristics of animals such as taxonomy or feeding habits. However, the detailed study of Van Wormhoudt and colleagues [54] provided important information in this regard. In that study, shrimps and crabs showed the highest activity among 40 species analyzed, while comparatively low activity in one carnivorous spiny lobster species. Yet, the study reported very few differences in the specific activities of the pure enzymes, suggesting that the catalytic features of α-amylases from crustacean decapods might be similar [54]. Thus, differences in activity among groups or species might be more related to the amount of enzyme synthesized and/or secreted into the digestive tract. Indeed, the α-amylase content of digestive gland of the carnivore crab *C. maenas* and of the carnivore-scavenger *Pagurus bernhardus* is about 0.1% of total proteins, whereas it was 1% in the omnivores *L. vannamei* and *Procambarus clarkii* [54]. However, little information is available on the regulation of transcription, synthesis, and secretion of α-amylases at the molecular level in crustaceans (See Section 6).

## 5. Alpha-Amylase Polymorphism

Alpha-amylase polymorphisms have been mostly studied in insects, mollusks, and higher vertebrates. Analysis of α-amylase activity of two α-amylase variants (AmyS and AmyF alleles) in *Drosophila* revealed that specific α-amylase activity is higher in in specimens possessing the S allele than in individuals with the F allele [107]. These isoenzymes differ in thermostability and kinetic characteristics [108], and their different activity affects the fitness of the different genotypes [109]. In some fly species, α-amylase activity differences are thought to be also connected to gene polymorphisms [110]. In chickens, a very distant group in respect to insects, the effects of α-amylase polymorphism on digestion capacities (e.g., changes in food conversion ratio) are also due to biochemical difference among isoforms [111]. However, in the oyster (*Crassostrea gigas*), the digestive α-amylase also exhibits a high level of polymorphism [112] influencing growth [113], but this is likely due to variation in the level of α-amylase gene expression rather than to functional enzymatic differences [114]. Alpha-amylase polymorphisms have also been thoroughly studied in humans, and both situations described above are known to occur. Human salivary α-amylase is encoded by AMY1 gene, which shows extensive copy number variations [115] and significantly affect individual salivary α-amylase amount and activity [116]. It has been suggested that such copy number variation of AMY1 is most likely an adaptation to diets rich in starch [115], although others have proposed that starch digestion may be not the major selective force [117] and that AMY1 copy number variation is a minor contributor to variation in salivary α-amylase expression and activity [118]. However, a recent study analyzed the genomes of a range of mammals and definitively found that the more starch a species had in its diet, the more α-amylase gene copies it harbored in its genome [119]. It is also known that salivary α-amylase is absent in pure carnivores mammals, whereas it is presents in some herbivores and many omnivorous [120].

Alpha-amylase polymorphism is less understood in decapod crustaceans. It was studied by electrophoresis in 40 species of decapods, with five or six isoforms in some species [54] and up to ten in some shrimps [13]. Conversely, only one or two isoforms occur in individual lobsters *P. argus* [24] and other crustaceans [54]. Although in an early study we found up to four forms of the enzyme in the lobster *P. argus*, nearly all individuals exhibited only one or two isoforms [18]. Thus, crustaceans with omnivorous feeding habits including all detritus, plants, and animals in their diet seem to have more α-amylase isoforms than carnivorous [18,54]. A recent study in the opportunistic feeder shrimp *Crangon crangon* also sustains this trend, and four putative α-amylase isoforms were identified, with two of them being the main forms of the enzyme [121]. Exceptions occur, as we recently found a single α-amylase form in the omnivorous crab *M. spinosissimus* in spite of high α-amylase activity [73].

One reason for α-amylase richness in some crustaceans is the presence of duplicated genes [45]. However, this cannot explain the totality of the isoenzymes observed. For instance, three α-amylase genes were found in the shrimp *L. vannamei* [45] but eight isoforms can be observed by electrophoresis [122]. Likewise, only one α-amylase gene is found in the lobster *P. argus* [24] although two isoforms are present in the digestive gland of some individuals [18]. It is clear that gene duplications (and maybe gene losses) have occurred in different crustacean lineages during evolution, as shown for mammals [119] and insects [123] driven by feeding habits, but other sources of polymorphisms remain poorly studied. In the case of the lobster *P. argus*, a single protein gives rise to two isoenzymes in some individuals by glycosylation but not by limited proteolysis [24]. The glycosylated form of the enzyme is the slower migrating form. Glycosilation is also the cause of several forms of the human salivary α-amylase [102,124]. It is still not clear why differences in the glycosylation pattern among human amylase isoenzymes occur, as this modification has no major effect on the activity of the enzyme [125]. Moreover, glycosilation was shown to have no effect on activity, optimum pH, or temperature in other amylases such as those of yeast, but to increase stability, decreasing sensitivity to inactivation by trypsin and high temperature [126], in agreement to the general notion that glycosylation aids in folding of the nascent polypeptide chain and in the stabilization of the mature glycoprotein [127].

The physiological significance of α-amylase polymorphism in decapods is poorly understood. Even in groups more deeply studied such as insects, where wide information supports the notion that several gene copies may increase dietary flexibility, sometimes the number of α-amylase gene copies cannot be clearly related to the diet as it may vary between species that share similar diets [5]. Alpha-amylase genotypes and differences in activity among isoforms are known to affect habitat and food choice in other crustaceans such as amphipods (*G. palustris*) [128,129] and isopods (*A. aquaticus*) [130]. In the shrimp *L. vannamei*, four single nucleotide polymorphisms (SNPs) were found in AMY2, but none were associated with body weight [131]. In the lobster *P. argus*, in vitro studies at our laboratory examined whether the three α-amylase phenotypes differed in digestion efficiency. Most individuals only exhibit the non-glycosylated α-amylase (isoenzyme of higher electrophoretic mobility), while others only have the glycosylated form or both [24]. Lobsters only exhibiting the glycosylated form of the α-amylase were the less common. For each carbohydrate substrate analyzed, we observed differences among phenotypes in their digestion efficiency. Interestingly, the most frequent isoenzyme, the non-glycosylated form, is the one of less digestion efficiency. Thus, α-amylase polymorphism in the carnivorous lobster population seems to be influenced by selective forces toward less carbohydrate digestion. These studies point to that the phenotype with lower digestion efficiency is favored at the population level. Mechanisms enabling long-term persistence of α-amylase polymorphisms in lobster and other crustaceans’ populations are unknown but they are likely to involve natural selection. Moreover, little information is available on the adaptive value of α-amylase polymorphism in crustaceans when faced with other environmental challenges. In this way, crabs *N. granulata* acclimated to 35 psu exhibited at least five bands with amylolytic activity, while crabs acclimated to 10 psu showed an additional amylolytic band of about 30 kDa, which correspond to a higher total α-amylase activity in this later group [10]. The authors claimed that whether differential expression/synthesis of α-amylase and/or posttranslational modifications is occurring upon acclimation to low salinity remains to be investigated.

## 6. Alpha-Amylase Regulation

Digestive enzymes synthesized in F cells of the digestive gland of crustaceans [132] are discharged into the gland lumen and then accumulated in its active form in the stomach. The synthesis of α-amylase and other digestive enzymes in crustaceans has been recently reviewed [35]. Studies in crustaceans have regularly reported high α-amylase activity in the gastric fluid of unfed animals. Indeed, α-amylase activity is higher in the gastric juice than in the digestive gland in the lobsters *Homarus gammarus* [57], *Jasus edwardsii* [133], and *P. argus* [18]; the crayfish *Macrobrachium rosenbergii*; as well as the shrimps *Penaeus monodon*, *P. indicus*, and *Metapenaeus monoceros* [134]. This indicates that both transcription and secretion are key regulatory point for α-amylase in crustaceans. However, little is known on the molecular mechanisms involved in the α-amylase regulation. An ecdysteroid-responsive α-amylase gene was identified in the crayfish *P. clarkii*, whose expression is down-regulated in digestive glands at 48 h after ecdysteroid induction [68]. Moreover, the crustacean hyperglycemic hormone (CHH) is able to stimulate α-amylase release from the digestive gland of the crayfish *Orconectes limosus* [135]. The vertebrate’s hormones, gastrin and secretin, are also able to exert the same effect in the gland of this crayfish [136], probably via cAMP [135], suggesting the presence of receptors for these hormones in the digestive gland of this crayfish. Interestingly, bilateral ablation of eyestalks of the crab *Eriocheir sinensis*, and thus the source of several neuropeptides, increased α-amylase activity in males, but not in females. The explanation that the author suggested was that eyestalk ablation speeded up the development of testis and consequently, males need to consume larger amounts of energy [137]. More research is needed to better understand these mechanisms at the molecular level. To date, most studies have been focused on the description of variations of the activity or transcription through ontogeny and molt cycle (see Section 6.1), or after feeding diets of a varied composition (see Section 6.2).

### 6.1. Development and Molt Stage

Variations in digestive enzyme activities reflect the maturation of the digestive system at early stages and later, changing physiological requirements as animals grow. Often, a clear relationship with shift in diet composition can be observed, while in other cases, contrasting results have been reported. In general, phytotrophic larval stages show an apparent predominance of trypsin content, while in carnivorous larvae a higher ratio of α-amylase to protease is observed [138]. However, variation occurs. For example, α-amylase activity is extremely low during carnivorous early larval stages of *M. rosenbergii* while increased sharply when the animal develop into an omnivore juvenile [139]. In the spider crab *Maja brachydactyla*, α-amylase showed a continuous enhancement of total activity through development, and zymograms revealed that α-amylase-active bands increased in number and intensity as development advanced [7]. Likewise, α-amylase activity in the predator larva of the lobster *H. americanus* increased slightly at the time of hatching and also during larval Stages I and II, achieving maximal activity among Stage V juveniles [103]. Conversely, in another crab, *S. serrata,* α-amylase activity enhanced through first stages of developments (i.e., zoea) but then gradually declined at more advanced stages [140], as also occurred in the crayfish *P. clarkii* [141]. Early shrimp larvae fed on phytoplankton and gradually incorporate zooplankton in their diets [142]. In the shrimps *Penaeus setiferus* and *P. indicus*, peak activities for all enzymes occurred during late zoeal or early mysis larval stages and later, α-amylase activity significantly increased during postlarval development [143,144]. In *L. vannameii*, 9 out of 16 unigenes enhanced their expression from nauplius to zoea contributing to a significant increase in activity [46]. However, contrasting results have been obtained in juveniles sharing similar feeding habits. For instance, the α-amylase importance in digestion seems to decrease as the omnivore anomuran crab *Aegla uruguayana* juveniles grown, while in the omnivore crayfish *Macrobrachium borellii* this trend is not evident [145]. In other omnivore crayfish, such as the redclaw *C. quadricarinatus,* α-amylase activity remains relatively constant in early juveniles but shows a great increase in larger animals [146].

In the carnivore lobster *P. argus* we found no trends in the relationship between specific α-amylase activity and size (in a range from 6 to 20 mm carapace length, i.e., from first post-pueruli to first juvenile stages) [18]. However, in juveniles and adults, there is a significant positive relationship between specific α-amylase activity and lobster size, suggesting that the capacity for carbohydrate digestion increases as lobsters grow [19] and fed on bigger prey items probably with a higher content of glycogen. Indeed, multivariate analysis suggested that in *P. argus* digestive enzyme activities appear to be strongly influenced by changes in diet [19]. Conversely to that found in *P. argus*, small *J. edwardsii* exhibit higher α-amylase activity than large specimens [34].

There were no clear shifts in the electrophoretic pattern of α-amylase through development and the molt cycle in lobster [18], indicating that regulation of activity is quantitative. Variations in the activity of digestive enzymes in the lobster *P. argus* resemble its foraging patterns through the molt cycle, and changes in activities are similar for almost all enzymes. After molt, α-amylase activity gradually increased to maximal levels at late intermolt (C4) and premolt (D). During late stage C, few glycogen granules are evident in the digestive gland of *P. argus*, but their number enhances during stage D both in the digestive gland as well as epidermis. This glycogen disappears some days after molt, likely used as a precursor for chitin formation [147]. In this scenario, α-amylase activity enhancement at late intermolt and during premolt might stimulates carbohydrate assimilation and formation of glycogen reserves. Our results agree with those obtained in other decapods such as *Palaemon serratus* [148], *Penaeus notialis* [149], *Farfantepenaeus duorarum* [150], *Macrobrachium tenellum* [151], and non-decapod crustaceans such as the amphipod *Gammarus fossarum* [152]. However, deviations have also been observed from this pattern. Alpha-amylase activity decreases from intermolt to premolt and then abruptly increased at molt and postmolt in the crab *Callinectes arcuatus* [153]. The interaction between molt stage and the environment on α-amylase activity has been poorly assessed. In the shrimp *L. vannamei*, specific activity of α-amylase is affected by the interaction between salinity and moult stages, resulting in highest values at stage C for low salinity and at D0 in high salinity [154]. Taking into account the variability of habitats of decapods, from a narrow to wide range of salinity, this is an issue that deserves further species-specific investigation.

### 6.2. Feeding Habits and Diet Composition

The ability of organisms to adapt to the characteristics of the diet to cover the requirements of certain nutrients has been documented in a wide variety of species, including crustaceans. This ability relies largely on variations in the activity levels of digestive enzymes. A positive correlation of α-amylase activity and dietary carbohydrates has been reported in very distant groups such as insects [155,156], mollusks [113], fish [157,158], dogs [159], and humans [115], and also relates positively with the amount of transporters necessary for their absorption at intestinal level [160]. In general, high amylolytic activity in herbivorous and omnivorous is accepted to result from adaptation to low energy food and low assimilation efficiency or as an adaptation to large amounts of dietary starch [54,161]. An early study comparing digestive enzymes of crustaceans with different feeding habits suggested that omnivores have more α-amylase activity than carnivorous species [161]. Much later, the most comprehensive assessment of α-amylase activity in crustaceans included 40 different species and confirmed that omnivorous crustaceans such as shrimps, crabs, and crayfish have relatively high α-amylase activity with respect to carnivorous species [54]. Other studies also reported that omnivorous crab species present high α-amylase activities [26]. In agreement, in a comparison among decapods with different feeding habits, the highest α-amylase to protease ratio was observed in adults of the omnivore shrimp *Macrobrachium australiense* and the lowest in mostly carnivores crabs *Portunus pelagicus* and *S. serrata* [64]. Also in this line, some herbivore crayfish exhibits higher α-amylase activity than omnivore shrimps [114]. However, few contradictory results have also been obtained. Alpha-amylase activity in adults of the omnivore shrimp *P. indicus* is higher than in other omnivore shrimp, *L. vannamei*, especially at high temperatures [59], suggesting a role of environmental temperature on this activity. Likewise, the association of α-amylase activity and diet was not clear in four land crabs species with detritivorous or omnivorous feeding habits [162]. In this regard, it is important to remark that several factors converge for the adaptation to a particular trophic level such as live history, metabolic rate, behavior, and other features of the digestive processes including food intake, mechanical digestion, retention time, and assimilation efficiency [27]. Moreover, digestive enzymes other than α-amylase often have a major role in carbohydrate digestion [27]. This is the case of enzymes that digest cellulose (endo-ß-1,4-glucanase, cellobiohydrolase, ß-1,4-glucosidase) and hemicellulose (laminarinase, lichenase, xylanase) in herbivore species such as land crabs, coincident with the higher level of these carbohydrates in their diets with respect to starch [27].

Moreover, α-amylase activity has been regularly reported in carnivorous crustaceans [18,26,34,55]. The relatively high α-amylase activity in spiny lobsters seems to contradict their limited metabolic use of carbohydrates [23,25,163], evident by the reduced activity of enzymes involved in both glycolysis and glycogen synthesis [23,25], although a recent study revealed that carbohydrate was the predominant energy substrate in 3-day fasted lobsters if previously fed a low (i.e., 40%) protein diet [164]. Yet, carbohydrates continue having a less important role as energy substrate after feeding, and even in fasted lobsters, if previously ingested a protein rich (i.e., 50%) diet [164]. Likewise, the high α-amylase activity in carnivorous larvae of the spider crab *Hyas araneus* does not correspond to the low carbohydrate content in its food and this was suggested to be a phylogenetic remnant from ancestor species with partly herbivorous larvae [165]. Interestingly, results in four closely related prickleback fishes showed that activity of α-amylase follows a pattern influenced more by phylogeny than by diet in these fishes [166] suggesting that this could be a common pattern.

Regarding the adaptation of crustaceans’ α-amylase to diet composition, juveniles exhibit more plasticity than larvae. In larvae and postlarvae of the shrimp *P. japonicus*, α-amylase was less affected by herbivorous or carnivorous feeding than other digestive enzymes such as trypsin [167]. Likewise, starch between 1% and 20% in feed had no influence on α-amylase activity in the shrimp *L. vannamei* larvae [168]. Conversely, in juveniles of other shrimp, *P. monodon,* α-amylase activity was higher in individuals fed wheat starch and sucrose-containing diets than in those fed diets containing potato or maize starch, dextrin, maltose, or glucose [169]. Also in juvenile of other omnivorous crustaceans, the crayfish *P. clarkii*, this activity enhanced with dietary corn starch levels [170]. The source of carbohydrate also affects α-amylase regulation in *L. vannamei* juveniles as this activity increased with corn starch in respect to that observed with soluble starch, amylopectin corn starch, or pregelatinized corn starch [168]. In addition, food also induced changes in the presence of different α-amylase isoforms in juvenile of this shrimp, with two major forms at specimens receiving a diet with 25% casein and only one in that feed diet with 40% casein [45]. This regulation appears to be at the transcription level [45]. These authors suggested that this regulation may be exerted by the level of casein in the diet, the ratio between protein and starch, or to a more complex mechanism, as it is also supported by studies in lobster [25].

In the carnivorous lobster *J. edwardsii*, α-amylase activity is higher when they ingest fresh mussel (with low carbohydrate content), while it decreases if they are fed 25% carbohydrate diets [171,172], indicating some capacity to regulate α-amylase activity depending of composition of food. In agreement, in the carnivorous lobster *P. argus*, we found an increase both in expression and activity when fed on fish muscle (~2% to 5% glycogen) with respect to diets with 30% starch, although this regulation is not affected by the source of starch [24]. These results demonstrated that carnivorous lobsters α-amylase respond differentially to natural diets and formulated feeds. Moreover, they suggest that lobsters are not able to regulate α-amylase expression and activity according to the source of carbohydrates in diet, which omnivore shrimps can do [168]. In a further study, we fed lobster with fresh fish or the three formulated diets only differing in carbohydrate content (6%, 20%, and 35%) and examined α-amylase expression and activity 24 h later. Differences in α-amylase gene expression were only found between animals fed with fresh fish and the 35% carbohydrate diet. Thus, lobsters were not able to fine-regulate α-amylase gene expression in spite of large changes in carbohydrate composition in diet (e.g., 6% to 35%) [25]. Therefore, it can be postulated that transcriptional regulatory mechanisms for α-amylase are not well developed in carnivorous lobsters, while retaining some ability to adapt α-amylase activity to very low carbohydrate diets. Our results in lobsters agree with the general notion that carnivorous species present low enzyme flexibility. Indeed, no significant differences were observed in α-amylase activity in digestive gland extracts from the shrimps *Artemesia longinaris* and *Pleoticus muelleri* with variation in starch inclusion in diet [173]. While phylogenetically distant from lobsters, these shrimps are also predators and fed mainly benthic fauna, although they may ingest detritus and vegetal material to a lesser extent. Recent results in the carnivorous lobster *P. argus* have shown that in addition to genome simplification, transcriptional regulatory mechanisms have been simplified, being more responsive to unknown general signals from diet (e.g., fresh food vs. formulated diet) than to specific carbohydrate levels. Secondly, while gene expression in the digestive gland is similar in lobsters ingesting fresh fish and formulated diets, lobster feeding on fresh fish exhibited a significantly higher activity in the gland [25]. This finding suggests that in addition to regulation at the transcription level, there is a regulation of amylase activity at the secretion level that is probably more important [25]. Future studies are required to broaden this issue.

## 7. Carbohydrate Digestibility

The susceptibility of starches to hydrolysis by α-amylases has been reviewed elsewhere [41]. In general, it depends on the content of amylose, which hinder digestion because the tight packaging of its structure [174] and to the formation of amylose-lipid complex [175,176,177]. Starch digestion also depends on the size of the granule because of its impact on the area available for enzyme digestion [178,179,180]. While α-amylases are responsible for much of the carbohydrate digestibility, especially those included in formulated feeds, few studies on carbohydrate digestibility have linked digestibility with α-amylase activity. Despite that, these studies provided important clues on the substrate preference for the α-amylases of different species.

### 7.1. In Vitro Digestibility

Currently several methods for evaluating the in vitro digestibility of feed have been developed as an alternative to costly and time-consuming in vivo digestibility tests. These assays provide a valuable approach to in vivo digestion processes [43]. Furthermore, as they are simpler and faster, they can be used to analyze a large number of raw materials, which is convenient for initial studies in species for which there is no previous information. This is the case of the spiny lobster *P. argus*, where in vitro digestibility was used [23,24] as a first approach to the digestion of carbohydrates in this species, and for subsequent designs of in vivo experiments. Using the “digestion in vials or Eppendorf tubes”, it was possible to compare, for the first time, the digestibility of 13 carbohydrate sources in the spiny lobster *P. argus* [23]. Native rice starch displayed the highest in vitro digestibility of all the carbohydrate substrates tested [23,181]. Other carbohydrates were also digested at a high rate such as gelatinized potato starch and gelatinized maize starch. Intermediate digestibilities were obtained for rice flour, wheat flour, potato starch, maize flour, glycogen, and maize starch. Finally, the lowest digestibilities were found for carbohydrates such as carboxymethyl cellulose, alginate, agarose, and agar, whose hydrolysis depends on other carbohydrases [27,57,74,133].

Nutritional studies in crustaceans such as *L. vannamei* [182], *J. edwardsii* [133], *H. gammarus* [57] also provided evidence of the high digestibility of native wheat starch. It is known that high digestibility of native wheat starch is due to the high amylopectin content (~80%) [32] of its A-type granules [183]. Maize starch was neither well digested in other crustaceans [184,185,186], including the spiny lobsters *J. edwardsii* [133,163]. Starch from maize has relatively small granules, but a high content of amylose and a polyhedral form, which are two factors that affect hydrolysis negatively [187]. In general, in vitro studies indicated similar substrate preferences of α-amylase among decapod crustaceans.

### 7.2. In Vivo Digestibility

An early study in the lobster *J. edwardsii* used the glycemic response as an indicator of carbohydrate digestibility [188] and another study in the same species reported that the use of wheat starch in diet formulations improves diet digestibility [163]. In a previous study we compared the in vivo digestibility of three carbohydrate source (wheat flour, rice starch, and maize starch) for the lobster *P. argus*, being the digestibility 90.7%, 81.4%, and 60.1% respectively [23]. In shrimp starch digestibility varies from 60% to 96% [182], while in other spiny lobsters changes from 59% (maize) to 91% (wheat) [163]. These variations depend not only on the features of the starch granules and the activity of α-amylase enzymes as discussed above, but also on the level of carbohydrate in diet, the ration size, and the throughput rate of the digesta [189]. In general, transit time varies in crustaceans from as little as 30 min in small copepods to over 150 h in larger lobsters [190]. The transit time is also affected by environmental factors such as temperature, salinity, and oxygen tension [190]. These results indicated that in vivo carbohydrate digestibility is depending of factors related to dietary carbohydrate (source, content, presentation, etc.) as well as environmental factors.

On the other hand, it has been shown that the type of carbohydrate ingested has a profound effect on intermediate metabolism, at least in *P. argus*, affecting the metabolism of carbohydrates, amino acids, and fatty acids [25]. Unlike other sources of carbohydrate, the use of wheat flour in the diet decreases the oxidation of amino acids while stimulating the use of fatty acids in energy metabolism. However, the ability of wheat flour for protein sparing effect from catabolic use directly through increased carbohydrate utilization via glycolysis is limited to a 20% inclusion level for lobster [25]. Most of the research on carbohydrate metabolism in crustaceans has been focused on dietary carbohydrate requirements and utilization, digestive enzymes, and immunity, with fewer studies on glucose transporters and glucoregulation [31]. In particular, there is a deficiency of research on glucose transporter proteins (except for the GLUT family), regulators involved in carbohydrate metabolism, and the role of different hormones [31]. The relationships among these factors and amylase enzymes are unknown in most species and also deserved further studies.

## 8. Summary and Open Issues

Decapod crustaceans are a very diverse group and have evolved to adapt to a broad variety of diets. However, α-amylases have been more thoroughly studies in herbivore and omnivore species, both from an evolutionary/ecological and applied (i.e., aquaculture) point of view, while information on α-amylases from carnivorous species is scarce. Diverse studies revealed that enzyme sequences and overall architecture is highly conserved among decapods. They are encoded by different genes in some omnivore species but there is evidence of gene and intron losses in at least one carnivore species. Recent evolutionary analyses revealed that positive selection might have occurred among distant lineages (e.g., herbivore crabs, omnivore decapods), with feeding habits as a selection force. Both bioinformatic (e.g., docking) and experimental studies would reveal which of these sites within amylase have truly evolved to better fit diet or environment of different crustacean groups. Some biochemical features of decapod α-amylases can be related with habitat or gut conditions, such as the effect of sodium, calcium, optimal pH, and temperature. However, less clear patterns are observed in their thermal stability and catalytic properties, although they all exhibit high activity toward native wheat starch. More studies on the substrate specificity of crustacean ∝-amylases would increase the understanding of their digestive physiology, and aid in the selection of carbohydrates for formulated diets. Although exceptions occur, omnivore decapods seem to have more α-amylase isoforms than carnivorous, but the number of genes does not totally explain this variation. At least in the carnivore lobster, with a single α-amylase gene, polymorphism arises by glycosylation. While α-amylase polymorphism is related to habitat and food choice in other crustacean groups such as amphipods and isopods, it physiological significance in decapods is poorly understood. In the carnivorous lobster, differences in digestion efficiency among α-amylase phenotypes were found, with less carbohydrate digestion being favored at the population level. There are also reports on the presence of specific isoenzymes induced by changes in environmental salinity, but again, the significance of this plasticity is not known. More studies on this issue would have important implications in the aquaculture sector, and specifically for farmed crustaceans with genetic selection programs. Molt cycle variations in α-amylase activity are rather similar among species, but through development, clear relationships with diet shifts can be observed in some cases and not in others. Omnivorous crustaceans such as shrimps, crabs, and crayfish have relatively high α-amylase activity with respect to carnivorous. Yet, contradictory results have also been obtained and high activity in some carnivores has been suggested to be a remnant trait from ancestor species.

Here we provided information sustaining that high enzyme sequence and overall architecture conservation do not allow high changes in activity, and that differences among species may be more related to number of genes and isoforms, and transcriptional and secretion regulation. Regarding the adaptation of crustaceans’ α-amylase to diet composition, juveniles seem to exhibit more flexibility than larvae, and there are reports on variation in α-amylase activity or number of isoforms because of the type of carbohydrate and its level in diets, especially in omnivore species. In the carnivorous lobster, however, no influence of the type of carbohydrate could be observed. Moreover, lobsters were not able to fine-regulate α-amylase gene expression in spite of large changes in carbohydrate composition in diet, while retaining some ability to adapt α-amylase activity to very low carbohydrate diets. Thus, while transcriptional and secretion regulation for decapod α-amylases have been reported, more mechanistic studies are needed. In this review, we raised arguments for the need of more biochemical and molecular studies on the α-amylases of less studied decapods groups, including carnivores which rely more on dietary protein and lipids, to broaden our view of α-amylase evolution and functional role across decapods crustaceans.

## Figures and Tables

**Table 1:** Conservation (i.e., identity) of the lobster *Panulirus argus* α-amylase cDNA sequence (GenBank accession no. LK937698, 1830 bp long) with respect to other α-amylases from decapod crustaceans and humans.

Group	Species	Accession No. Genbank	Identity (%)	Nucleotides (pb)
Brachyurans	*Eriocheir sinensis*	KU301756.1	75.6	1663
	*Helice tientsinensis*	MN964184.1	75.39	1527
	*Neohelice granulata*	KU531567.1	75.04	1637
	*Macrophthalmus pacificus*	MN964194.1	74.34	1533
	*Gelasimus borealis*	MN964240.1	76.19	1533
	*Metopograpsus quadridentatus*	MN964203.1	76.25	1533
	*Parasesarma pictum*	MN964222.1	76.22	1533
	*Parasesarma affine*	MN964213.1	76.09	1533
	*Chiromantes dehaani*	MN964164.1	75.36	1533
	*Sesarmops sinensis*	MN964231.1	75.21	1215
	*Calappa philargius*	MN964146.1	69.98	1533
	*Charybdis japonica*	MN964155.1	76.32	1533
	*Scylla olivacea*	GDRN01093055.1	75.51	1715
	*Portunus trituberculatus*	MN964137.1	74.37	1533
Penaeids	*Marsupenaeus japonicus*	KJ147432.1	77.95	1651
	*Penaeus monodon*	KU308415.1	66.34	2465
	*Litopenaeus vannamei*	KM077131.1	66.17	2358
Carideans	*Crangon crangon*	MH055762.1	66.43	2175
	*Macrobrachium rosenbergii*	KM886337.1	67.6	2282
Astacids	*Astacus leptodactylus*	KF954216	65.69	2250
	*Homarus americanus*	XM_042364069	67.45	2434
	*Procambarus clarkii*	MF688642.1	67.17	2138
Human	*Homo sapiens* *nlknln*	M24895.1	66.85	1612

**Table 2:** Biochemical features reported for α-amylase in decapod crustaceans. Information from few species of other crustaceans and other taxa was included for comparative proposes.

	*Km*	Number of Isoforms	MW (kDa)	Opt. pH	Opt. Temperat.(°C)	NaCl	Ca^2+^	References
**Crustaceans**								
**Lobsters**								
*Panulirus argus*	0.36 mM *	2	55.5	5–6	50	0.3 mM	↑ up to 25 mM	[24]
*Panulirus japonicus*				4.9				[53]
*Panulirus interruptus*		1						[54]
*Jasus edwardsii*				5.5				[34]
*Homarus americanus*			41	5.2–5.5		+ (0.05–0.1 M)	−	[55,56]
*Homarus gammarus*				4.8				[57]
*Thenus orientalis*				5.0–5.8				[58]
**Shrimps**								
*Litopenaeus vannamei*		7–10		7–8	40–50		↑ up to 1 mM ↓ >5–10 mM	[13,54,59]
*Litopenaeus schmitti*		8		7	40		↑ up to 1 mM ↓ >5–10 mM	[13]
*Farfantepenaeus subtilis*		9		7.5	45		↑ up to 1 mM ↓ >5–10 mM	[13]
*Farfantepenaeus californiensis*				7.5	30–40	0.01 M	−−	[60]
*Penaeus monodon*		2		5.4–7			++	[61]
*Penaeus japonicus*				6.8	40		↑ up to 1 mM	[62]
*Penaeus indicus*		1		6.6–7–8	37			[54,59,63]
*Penaeus esculentus*				7				[64]
*Penaeus plebejus*				5				[64]
*Metapenaeus bennettae*				7				[64]
*Metapenaeus monoceros*				7	40			[63]
*Macrobrachium australiense*				5				[64]
*Macrobrachium lamarrei*	9.0 × 10^−^^2^%			6.5	50	+		[65]
*Palaemon elegans*		7	29–78					[54,66]
**Crayfish**								
*Orconectes virilis*				5.9–6.3		+		[67]
*Procambarus clarkii*		1	55	5.8	55.1			[54,68,69]
*Cherax quadricanatus*				6			↑ up to 15 mM	[70]
*Cherax albidus*		4	38, 44, 49, 55	6.5	25			[71]
*Astacus leptodaytylus*		6						[54]
**Crabs**								
*Carcinus maenas*	0.22%	2	30–35	6.8	40	++	++	[54,72]
*Maguimithrax spinosissimus*		1	40	5–6.5	40–60	−	↑ up to 2.5 mM	[73]
*Scylla serrata*				6.5–7	50			[64,74]
*Neohelice granulata*	1.24 mg/mL	5	26–36		30–40	↑ up to 1.5 M ↓ >1.5 M	++	[10]
*Cytograpsus angulatus*	0.11 mg/mL	2	31, 38	5–7	30			[75]
*Portunus segnis*	7.5 mg/mL		45	7.5	45–65		−	[76]
*Portunus pelagicus*				6.5				[64]
*Maja brachydactyla*		4	27–68					[7]
*Uca minax*		1		7.3		0.075 M		[77]
*Uca pugnax*		1		7.3		0.1 M		[77]
*Uca pugilator*		1		7.3		0.1 M		[77]
*Cancer borealis*				7.0				[6]
*Cancer irroratus*				7.0				[6]
**Isopods**								
*Asellus aquaticus*	10.4 mg/mL		≈70	5.4–5.8	60	↑ up to 1 M ↓ >1 M		[78]
**Amphipods**								
*Gammarus* *palustris*	0.045% 0.042%	5	50–69.4	7.5	30	↑ up to 8 mM		[79]
**Copepods**								
*Acartia clausi.*	4.5 mg/mL		44	6–6.7	40	↑ up to 0.1 M		[80]
*Heliodiaptomus viduus*	1.96 µg/mL^−^^1^		50	5.5–6	30		+	[81]
**Other arthropods**								
Scorpion *Scorpio maurus*		1	59	7	50	↑ up to 0.2 M	↑ up to 3 mM	[82]
Coleoptera, *Morimus funereus*	0.043%		33	5.2	45	↑ up to 0.2 M	↑ up to 0.1 mM ↓ >0.1 mM	[83]
Coleoptera, *Rhyzopertha dominica*	0.098%		52	7.0	40	+	+	[84]
Cockroach, *Periplaneta americana*	0.50%		60	5.6	55			[85]
**Nematods**								
Helminth, *Ascaris suum*		2	74, 83	7.4	40–50		↑ up to 0.5 mM	[86]
**Anelids**								
Earthworm, *Eisenia fetida*		2	63.8, 64	5.5	45, 35			[87]
**Mollusks**								
Sea hare, *Aplysia kurodai*	0.37 mg/mL 1.42 mg/mL	2	59, 80	5.5–6.5	40, 55		↑ up to 10 mM	[88]
Bivalve, *Mytilus galloprovincialis*		2	66	6.5	35–40	+	↑ up to 15 mM	[89]
Bivalve, *Haliotis discus discus*			54	6.5	50		↑ up to 2 mM	[90]
Bivalve, *Haliotis discus channai*		2	58, 82	6.1–6.7	30			[91]
Gastropod, *Concholepas concholepas*				7	50	++	++	[92]
**Echinoderms**								
Sea urchin, *Strongylocentrotus nudas*	2.28 mM			6.8		+		[93]
Sea urchin, *Anthocidaris crassispina*	2.1 mM			6.9				[94]
**Fish**								
Medaka, *Oryzias latipes*	1.18 mg/mL			7.12	49	↑ up to 0.2 M		[95]
Seabream, *Sparus aurata*		1	100	7-8	40–45	↓ >0.05 M		[96,97]
Turbot, *Scophthalmus maximus*				7	35–45	↓ >0.05 M		[96]
Redfish, *Sebastes mentella*				4–4.5	35–45	++		[96]
Mullet, *Chelon labrosus*				8	40			[98]
**Mammals**								
Porcine PPAI	135 mg/mL	2	55.4	7.3				[99]
Human Pancreatic	1.15 mM * 0.15 mg/mL		56	6.1				[100,101]
Human Salivary	2.22 mM *		56	5.9		↑ up to 0.3–0.4 M	↑ up to 4–5 mM	[100,102]

* CNP-G3 as the substrate; all other data obtained with starch. + Positive effect reported on amylase activity. − Negative effect reported on amylase activity.

**Table 3:** Available protein sequences of decapod crustaceans α-amylases in UniProt Database (https://www.uniprot.org/), accessed on 10 May 2021.

Group	Species	Uniprot Code	Length (aa)	Note
Brachyurans	*Eriocheir sinensis*	A0A173DQD0	517	
	*Helice tientsinensis*	A0A6G9W2W5	509	Fragment
	*Neohelice granulata*	A0A1L6BX60	439	Fragment
	*Macrophthalmus pacificus*	A0A6G9W2X5	511	Fragment
	*Gelasimus borealis*	A0A6G9W3V1	511	Fragment
	*Metopograpsus quadridentatus*	A0A6G9W6B4	511	Fragment
	*Parasesarma pictum*	A0A6G9W466	511	Fragment
	*Parasesarma affine*	A0A6G9W6C2	511	Fragment
	*Chiromantes dehaani*	A0A6G9W2T8	511	Fragment
	*Sesarmops sinensis*	A0A6G9W480	405	Fragment
	*Calappa philargius*	A0A6G9W4A8	511	Fragment
	*Charybdis japonica*	A0A6G9W484	511	Fragment
	*Scylla olivacea*	A0A0P4W0X7	517	
	*Portunus trituberculatus*	A0A6G9W4W3	511	Fragment
Penaeids	*Marsupenaeus japonicus*	X2KWV9	512	
	*Penaeus monodon*	A0A172GH45	724	
	*Litopenaeus vannamei*	A0A076L7X4	724	
Carideans	*Crangon crangon*	A0A2Z4BXI3	724	
	*Macrobrachium rosenbergii*	A0A0H3WET4	706	
Astacids	*Astacus leptodactylus*	A0A120GV93	696	
	*Procambarus clarkii*	A0A2Z5HVE6	713	Fragment
*Palinuridae*	*Panulirus argus* *nlknln*	A0A0G4DIJ9	513	

## Data Availability

Not applicable.
